# Understanding the Fight Against Resistance: Hospital-Acquired Methicillin-Resistant Staphylococcus Aureus vs. Community-Acquired Methicillin-Resistant Staphylococcus Aureus

**DOI:** 10.7759/cureus.8867

**Published:** 2020-06-27

**Authors:** Nicholas Tsouklidis, Rajat Kumar, Stacey E Heindl, Ravi Soni, Safeera Khan

**Affiliations:** 1 Medicine, California Institute of Behavioral Neurosciences and Psychology, Fairfield, USA; 2 Health Care Administration, University of Cincinnati Health, Cincinnati, USA; 3 Medicine, Atlantic University School of Medicine, Gros Islet, LCA; 4 Ophthalmology, California Institute of Behavioural Neurosciences and Psychology, Fairfield, USA; 5 Medicine, Avalon University School of Medicine, Willemstad, CUW; 6 Neurology, California Institute of Behavioral Neurosciences and Psychology, Fairfield, USA; 7 Internal Medicine, California Institute of Behavioral Neurosciences and Psychology, Fairfield, USA

**Keywords:** ha-mrsa, ca-mrsa, staphylococcus, staphylococcus aureus, resistance, hospital acquired mrsa, community acquired mrsa, mrsa prevalence, sccmec, pvl gene

## Abstract

Since the identification of Staphylococcus (S.) aureus, penicillin was exclusively used to combat its disastrous toxic effects. Shortly thereafter, resistant strains arose, which were no longer susceptible to penicillin or methicillin treatments. These strands were later identified as methicillin-resistant Staphylococcus aureus (MRSA). Two particular MRSA strands that are discussed below are the hospital-acquired methicillin-resistant Staphylococcus aureus (HA-MRSA) strands and the community-acquired methicillin-resistant Staphylococcus aureus (CA-MRSA) strands. Despite arising from a single bacterium, S. aureus, each of these two strands possesses quite different resistance and virulence factors. These differences contribute to the type of population in which they affect, their ability to resist traditional treatment approaches, and their overall morbidity and mortality rates. We explore these differences by reviewing several review articles published on various reputable scientific online databases. Findings include sources from studies conducted in the United States, China, Nepal, and Uganda, ranging from 2006 to 2019. These resistance and virulence factors, the Staphylococcal cassette cartridge mecA resistance gene (SCCmec) and the Panton-Valentine Leukocidin toxin gene (PVL), were identified and isolated in each of these studies in order to appreciate similarities and differences in how they impact human beings.

## Introduction and background

Staphylococcus aureus is a native bacterium to normal human flora and commonly found in the environment. In the majority of healthy individuals, S. aureus is located on the skin and mucous membranes (most often, the nares). In a healthy individual with intact skin membranes, this bacterium does not usually cause infection, however, once dissemination of S. aureus occurs in the bloodstream or internal soft tissues, potentially life-threatening infections may arise [1].

To better understand the differences between hospital-acquired methicillin-resistant Staphylococcus aureus (HA-MRSA) and community-acquired-MRSA and their degree of resistance, an analysis of their genetic and structural configuration must be explored. It is these resistance genes, virulence factors, and toxins that aid in further explaining the resistance Staphylococcus aureus expresses against most traditional therapeutic agents, thus contributing to the increased morbidity and mortality of patients [2-3]. This resistance makes Staphylococcus aureus one of the leading causes of nosocomial infections [4]. Staphylococcus aureus contributes to a variety of roles in disease. The diseases include but are not limited to skin infections, food poisoning, bone infections, bacteremia, and infections of implanted devices.

Staphylococcus aureus was first identified by the German scientist, Friedrich Julius Rosenbach in 1884, however, it was not until the 1930s when enzyme testing was used to detect a Staphylococcal infection caused by the bacteria’s ability to generate coagulase. Physicians began diagnosing and treating Staphylococcus aureus using this method and penicillin. Prior to 1940 and the use of penicillin, 75% of those infected by Staphylococcus aureus died. Unfortunately, by the end of the 1940s, a resistant strain had emerged resulting in unsuccessful treatment of Staphylococcus aureus by traditional penicillin [5]. Today, this resistant strain is expressed in hospital settings, HA-MRSA, as well as in the general population, or communities, CA-MRSA.

A few important genetic elements contributing to a better understanding of MRSA resistance include the presence of Staphylococcal cassette cartridge mecA resistance gene (SCC*mec*), as well as the Panton-Valentine Leukocidin toxin gene (PVL). There are various subtypes and categories to each of these genetic components, which contribute to the different virulence patterns and resistance characteristics of both HA-MRSA and CA-MRSA. Methicillin resistance is mediated by the mec operon, a component of the SCCmec family of genetic elements [6]. The PVL toxin gene is responsible for essentially converting a host’s tissues into nutrients necessary for Staphylococcus aureus to thrive. This exotoxin does this by destroying local leukocytes and other host defense cells in order to express itself as a virulent superantigen [7]. While there are many other exotoxins and virulence factors expressed by Staphylococcus aureus, by understanding the mechanisms of action of both SCCmec and PVL, and their expression, we will better understand how various strands of Staphylococcus aureus impact humans pathologically depending on the type of setting in which the bacteria is acquired, as well as the approach to successfully treat various conditions based on the specific Staphylococcal aureus strand in question.

This article aims to explore the similarities and differences between two distinct strands of MRSA in hopes to improve patient morbidity and mortality rates in the future. By comparing and understanding the resistance factors and toxins expressed by HA-MRSA and CA-MRSA, physicians and healthcare workers can improve MRSA incidence and recurrence rates by appreciating each strand's mechanism of infectivity and avoiding the root causes of resistance, ultimately leading to successful and cost-effective treatment. While recognizing HA-MRSA and CA-MRSA are both derivatives of a single bacterium, Staphylococcus aureus, they each possess quite different genetic components allowing them to impact humans differently as well as resist traditional medicine.

## Review

Methods

PubMed, ResearchGate, Antimicrobial Agents and Chemotherapy, Google Scholar, BioMed Central, and Medscape online databases were exclusively and systematically accredited for the collection of corresponding data. Results yielded 35 scientific papers, all composed in English. Of those 35 papers, specific inclusion and exclusion criteria were applied. After the application of the criteria and elimination of abstract reviews, 18 scientific papers were included in our final review. Of those 18 papers, all met the quality specifications and were peer reviewed.

Inclusion and exclusion criteria

All selected scientific papers were written in English and included data collected and reviewed from 2006-2020. All cases of HA-MRSA and CA-MRSA were confirmed using microbiological methods, including bacterial cultures obtained from the bloodstream, respiratory tract (sputum collection, bronchial alveolar lavage) skin and abscess samples, vaginal samples, and stool collections. Confirmed cases and interpretations of drug susceptibility strictly followed the Clinical Laboratory Standards Institute (CLSI) guidelines. Drugs that were included in studies included erythromycin, oxacillin, penicillin, vancomycin, clindamycin, TMP/SMX, nitrofurantoin, ciprofloxacin, gentamicin, tetracycline, levofloxacin, rifampicin, linezolid, tigecycline, and quinupristin/dalfopristin. Papers excluded lacked a sufficient collection of patient history and any history of prior comorbidities.

Results

Of the four selected research papers, all four confirmed the suspicion of a specific SCCmec subtype and PVL gene present in either MRSA strain in question, which contributes to that particular strain's pattern of resistance. Two of the studies went one step further to describe cross-mixing of HA-MRSA strands within a community, which was once exclusively thought to be dominated by CA-MRSA strains [8-9]. This is crucial data necessary when considering medication alternatives for resistant strains in such a varying incidence and prevalence rate, particularly in a bacterium that is capable of genetically adapting to traditional medication regimens [10-17]. In one study conducted in China, both CA-MRSA and HA-MRSA were 100% resistant to penicillin and oxacillin and 100% susceptible to vancomycin and linezolid. A study in Nepal concluded that isolating and identifying a positive PVL toxin may be a sufficient enough method to determine whether a particular MRSA strand is acquired through a hospital setting or within the community [18]. Table 1 organizes the studies used, which met inclusion criteria along with conclusions drawn from them.

**Table 1:** Description of selected studies that met inclusion criteria for this review HA-MRSA: hospital-acquired methicillin-resistant Staphylococcus aureus; CA-MRSA: community-acquired methicillin-resistant Staphylococcus aureus; PVL: Panton-Valentine Leukocidin

Study	Location	Study Period	Samples	Conclusion
Huang, et al. [8]	United States	2003-2004	283 MRSA samples, 127 (45%) were of CA-MRSA origin	CA-MRSA has disseminated into hospital systems and has most likely cross-mixed with hospital strands
Kateete, et al. [9]	Uganda	2011 (February-October)	742; 140 of 742 were S. aureus. 42 of 140 were MRSA	HA-MRSA strands were found to exist in the general population amongst eastern Ugandan children
Peng, et al. [15]	China	2012-2017	835; 80% HA-MRA, 20% CA-MRSA	Both CA-MRSA & HA-MRSA were 100% resistant to Penicillin and Oxacillin treatments, as well as 100% susceptible to vancomycin, linezolid, and tigecycline. CA-MRSA showed 94% susceptibility to ciprofloxacin while HA-MRSA showed only 14% susceptibility to ciprofloxacin
Bhatta, et al. [18]	Nepal	2012-2106	400; 139 MRSA (90% of CA-MRSA were PVL+)	PVL was a sufficient method of distinguishing CA-MRSA from HA-MRSA

Limitations

Several studies and clinical trials lacked thorough patient history information, which did not give a clear picture of the comorbidities patients may have endured through along with MRSA-induced skin and tissue infection.

Discussion

MRSA

One-third of the general population harbors S. aureus in the throat, nares, axilla, rectum, groin, and perineum, with the majority of MRSA cases arising from the nares and throat. Risk factors for MRSA colonization in the United States included low-income diabetic females above the age of 60. The only risk factor identified in US males included exposure to healthcare [8]. Other risk factors included intravenous drug use, chronic illness, and exposure to outpatient clinics.

MRSA infection continues to be a major healthcare complication worldwide. Staphylococcus aureus possesses the ability to disseminate in the bloodstream, leading to bacteremia, ultimately exhibiting high rates of morbidity and mortality. This dissemination leads to complications such as sepsis and infective endocarditis. Of these Staphylococcus aureus bacteremia cases, poorer outcome-related cases were associated with MRSA infection rather than methicillin-sensitive Staphylococcus aureus (MSSA) [11]. The primary contributing factor to a poor outcome and high morbidity and mortality rate amongst patients with MRSA infection can be directly attributed to the unique structural and genomic composition of Staphylococcus aureus, as well as the toxins and gene products expressed by specific strands of Methicillin-resistant* *Staphylococcus aureus*.* The two strands of MRSA discussed in this review are hospital-acquired MRSA and community-acquired MRSA, which despite deriving from a single common bacterium, differ greatly in the population that they affect, the toxins and virulence factors that they each produce, and their infectivity and resistance levels. One way in which microbiologists determine MRSA strains from MSSA strains is by the polymerase-chain-reaction (PCR) method of testing, which can be performed on samples taken from blood cultures as well as nasal or wound swabbing, aimed at targeting the MRSA-specific SCCmec gene. The second gene of interest, the Panton-Valentine Leukocidin toxin gene (PVL), is also of great importance when discussing MRSA resistance and can be identified by enzyme-linked immunoassay (ELISA), rapid monoclonal antibody test or by PCR testing. Treatment, however, must be initiated many times prior to confirming the results due to the toxic nature and rapid progression of Staphylococcal diseases as well as the limited testing availabilities at many hospitals. For this reason, exploring the MRSA resistance pattern is crucial in identifying highly effective treatment regimens.

Resistance Factors

When discussing MRSA infections, the primary concern for health care workers is overcoming Staphylococcal resistance factors by prescribing an antibiotic efficient enough to penetrate MRSAs' genomic composition and effectively eliminate the spread of this bacterium. To do so, scientists and microbiologists must identify and isolate two specific genes: SCCmec and PVL.

Staphylococcal cassette chromosome mec (SCCmec) is a defining feature of MRSA. SCCmec is an element of the genomic composition of MRSA, which is responsible for the determinant of beta-lactam resistance encoded by the mecA gene, which encodes the penicillin-binding protein 2a. Resistance occurs due to the acquisition and insertion of this SCCmec gene, composed of a mec gene complex and a ccr gene, into the chromosomal composition of certain susceptible Staphylococcal strains. SCCmec is a highly diverse gene classified into several various types and subtypes [10,12-13]. In 1960, methicillin was introduced to combat infections caused by bacteria that produce beta-lactamase, an enzyme produced by certain bacteria to resist and oppose the mechanism of beta-lactam antibiotics. Roughly one year later, the first strains of MRSA emerged, and all expressed SCCmec elements within their chromosomes. To date, eight SCCmec types have been identified for Staphylococcus aureus, each type slightly different in the contents of the SCCmec elemental components and their respective molecular sizes.

Panton-Valentine Leukocidin (PVL), encoded by two genes-lukS-PV and lukF-PV, is considered one of the most important cytotoxins produced by certain strains of Staphylococcus aureus. Sir Philip Noel Panton and Francis Valentine named this gene due to its soft tissue infections emerging in 1932. PVL’s mechanism of infectivity is directly due to its ability to induce pores within membranes of cells and further proceed to lyse white blood cells and prevent the body from establishing a defense mechanism to combat the bacterium’s toxic effects. According to expert analysis from the 2018 European Society for Pediatric Infectious Diseases (ESPID) symposium in Malmö, Sweden, the four most feared syndromes suggestive of PVL-positive Staphylococcus aureus include S. aureus pneumonia preceded by an influenza-like syndrome resulting in hemoptysis and a 50% mortality rate within the first few days, severe osteomyelitis, osteomyelitis with deep vein thrombosis, and osteomyelitis with septic shock [14]. A comparative analysis of CA-MRSA and HA-MRSA characteristics can be appreciated in Table 2 below.

**Table 2:** Differentiating characteristics of HA-MRSA and CA-MRSA HA-MRSA: hospital-acquired methicillin-resistant Staphylococcus aureus; CA-MRSA: community-acquired methicillin-resistant Staphylococcus aureus

	CA-MRSA	HA-MRSA
At-Risk Populations	Children, prisoners, homeless, homosexual males, soldiers, intravenous drug users, general population	Healthcare facility residents, diabetics, hospitalized patients, ICU patients
SCCmec subtype	IV, V	I, II, III
Resistant against	Beta-lactam drugs (oxacillin, penicillin), erythromycin	Usually multidrug resistance, tend to be susceptible to TMP-SMX, macrolides, tetracyclines
PVL toxin	Present in >95% of cases	Rare (5%)
Clinical Affiliation	Post-influenza necrotizing pneumonia, osteomyelitis	Nosocomial pneumonia, Catheter-acquired UTI, Bacteremia
Discovered	1980s	1961

In a study conducted from 2012 to 2017 in a Chinese tertiary hospital, 835 MRSA isolates were tested for the presence of these virulence factors [15]. Of the 835 MRSA isolates, 175 were CA-MRSA isolates, and 660 were of HA-MRSA origin. In 53.1% of CA-MRSA isolates, lukSF-PV positive genes were identified as well as all but one isolate containing SCCmec IV or V subtype genes. In contrast, HA-MRSA isolates were lukSF-PV negative in 88.2% of isolates.

In the first-ever study 2012 to 2016 study conducted at the microbiology laboratory of Manipal Teaching Hospital, Pokhara, Nepal, a total of 400 isolates had been obtained and tested for methicillin resistance by PCR methods. Of these 400 samples, 139 of them tested positive for MRSA. In this study, drug susceptibility was concluded by comparing PVL-positive and negative MRSA isolates, along with the reliability of PVL testing in identifying differences amongst HA-MRSA and CA-MRSA strands. Amongst the 139 MRSA samples acquired, 79 (57%) were PVL-positive. In 90% of CA-MRSA strands, PVL-positive isolates were identified and collected from pus samples. None of the HA-MRSA strains isolated contained PVL-positive qualities. This study further determined that antibiotic resistance was higher amongst specimens containing PVL-negative isolates when compared to PVL-positive isolates. It was determined statistically significant to assume PVL may be a sufficient indicator when comparing CA-MRSA to HA-MRSA [18].

Resistance Profiles of Hospital-Acquired MRSA and Community-Acquired MRSA

In reference to the 835 MRSA isolates isolated from a Chinese tertiary hospital between the years of 2012 and 2017, all specimen samples reported resistance to beta-lactam category antibiotics (oxacillin and penicillin). The majority of CA-MRSA strains showed susceptibility and sensitivity to vancomycin, linezolid, tigecycline, quinupristin/dalfopristin, ciprofloxacin, gentamicin, rifampicin, and nitrofurantoin. CA-MRSA strains, however, were resistant to clindamycin and erythromycin. One-hundred percent of HA-MRSA isolates were sensitive to vancomycin, linezolid, tigecycline, and quinupristin/dalfopristin. Less than 10% of HA-MRSA strands were susceptible to clindamycin.

Amongst the CA-MRSA strands, the predominant resistance factor subtype was discovered to be SCCmec IVa, which accounted for 70% of the 175 CA-MRSA isolates. Approximately 15% of CA-MRSA isolate samples contained SCCmec subtype V and subtype III in 10%. The majority of HA-MRSA isolates possessed subtype III (58%) and subtype II (25%). Interestingly, SCCmec subtype III identified in CA-MRSA strains showed more resistance to antibiotics, similar to those showing resistance in the HA-MRSA isolates. The HA-MRSA isolates carrying SCCmec subtype IVa exclusively showed resistance to oxacillin, penicillin, and erythromycin. Table 3 depicts the results of this particular study.

**Table 3:** HA-MRSA and CA-MRSA susceptibility/resistance profiles based on a 2012-2017 study conducted at a tertiary Chinese hospital HA-MRSA: hospital-acquired methicillin-resistant Staphylococcus aureus; CA-MRSA: community-acquired methicillin-resistant Staphylococcus aureus Source: [15]

Antimicrobial Susceptibility and Resistance: CA-MRSA vs. HA-MRSA	CA-MRSA (Susceptibility %) N=175	HA-MRSA (Susceptibility %) N=660	CA-MRSA (Resistance %)	HA-MRSA (Resistance %)
Penicillin	0	0	100	100
Oxacillin	0	0	100	100
Erythromycin	17	15	78	82
Ciprofloxacin	94	14	6	86
Tetracycline	82	17	15	82
Clindamycin	11	8	89	90
TMP-SMX	89	40	10	61
Rifampicin	92	69	3	30
Gentamicin	93	34	6	66
Nitrofurantoin	90	82	6	18
Vancomycin	100	100	0	0
Levofloxacin	70	42	29	58
Linezolid	100	100	0	0
Tigecycline	100	100	0	0
Quinupristin/Dalfo	99	100	0	0

From 2003 to 2004, a retrospective case series study comparing CA-MRSA and HA-MRSA was conducted in Sacramento, California. A total of 328 patient cultures were studied for over six months. Of the 328 samples, 283 of them tested positive for MRSA-positive isolates. Of those 283 samples, approximately 55% of them (156) fit the criteria of HA-MRSA and 45% of these samples (127) were classified as CA-MRSA. One significant observational difference noticed amongst the two categories of MRSA was the site of infection. Most commonly, skin and soft tissue sites were greatly affected in patients of both groups, specifically 61% amongst the HA-MRSA group and 86% in CA-MRSA patients. Diabetes, chronic renal insufficiency, and cancer were the most common underlying comorbidities observed amongst the HA-MRSA patient samples. When evaluating the CA-MRSA samples, nearly 50% of these patients were intravenous drug users (IDU), and only 18% of the HA-MRSA samples were of IDU. In this same study, it was determined that while CA-MRSA had emerged in this Sacramento, California community, this strand was mostly isolated in soft tissues and skin. The presence of CA-MRSA did not extend patients’ time or prolong their hospital stay. Most cases were responsive to incision and drainage followed by outpatient oral medications. CA-MRSA proved to be susceptible to various oral medications, including ciprofloxacin and clindamycin. Interestingly enough, erythromycin resistance in Sacramento patients was shown to be close to 93% while other communities in previous studies of Alaska and San Francisco showed lower resistance rates of 69% and 61% [8]. Another quite interesting observation from this study showed that while most HA-MRSA strands affected individuals with prior comorbidities, and CA-MRSA presented with more soft tissue and skin manifestations particularly amongst IDU, analyzing the virulence factors of S. aureus strands often resulted in cross-mixing. In other words, several HA-MRSA isolate resistance and virulence factors, while initially believed to be exclusive to HA-MRSA, were identified in several HA-MRSA strands. This event supports the fact that CA-MRSA has not only been identified in the hospital setting, but it has also managed to adopt several multi-resistant genes from the hospital-acquired MRSA strains. This issue poses challenges in determining the best course of treatment for patients who are suffering from comorbidities and ultimately leading to a prolonged hospitalization period and potential increases in mortality. Since 2019, in the United States, the incidence rates of MRSA infection ranges from 7%-60%. This number has steadily increased since the infection’s initial discovery and isolation in 1961 [16-17].

A 2011 study conducted in Uganda was aimed at focusing on MRSA carriage rates amongst children in eastern Uganda, as well as to determine the possibility of coexistence between HA-MRSA and CA-MRSA strands. A total of 742 nasopharyngeal samples were collected from healthy children less than five years of age in rural eastern Uganda and were processed for MRSA. Of the 742 samples, approximately 19% or 140 tested positive for MRSA, 95% of which had multidrug resistance. SCCmec elements expressed in these samples included subtype IV in 40% of the MRSA samples, as well as subtype I in 38% of MRSA samples. Given the understanding of which SCCmec subtypes are associated with each specific MRSA strand, this study was able to determine that in a healthy population of children in an eastern Ugandan community, both HA-MRSA and CA-MRSA strands were identified and isolated. While initially it was believed that the two strands were exclusive to their site of origin, this study demonstrated coexistence in a single community. This raises concerns for multidrug resistance of MRSA infections within the community [9].

## Conclusions

MRSA can be identified and isolated based on the presence or absence of specific virulence factors and toxins. These virulence factors, specifically SCCmec, include subtypes specific to HA-MRSA (I, II, III) and CA-MRSA (IV, V). Another method for determining the origin of the MRSA strand depends on the presence of PVL-positive toxin, seen in over 97% of CA-MRSA strands, or the complete absence of PVL demonstrated in HA-MRSA. By correctly identifying these factors, a proper treatment approach can specifically target each strand in a susceptible manner to eradicate an MRSA infection. Proper measures must be taken to not only prevent individuals from acquiring MRSA disease but to also prevent recurrence and cross-mixing of the two strands. By focusing on resistance factors and virulence factors, health care workers can appreciate the intricate genomic and molecular compositions of these two strands, identify any cross-mixing of these strands as witnessed in patients during the Sacramento, California, and Uganda studies, and better strategize a medicinal approach to eradicating MRSA infection. Other factors to consider include improving the hygienic measures taken by people daily and within the hospital setting. Hand washing, proper disposal of hazardous waste, removal of unnecessary catheters, and patient education against drug use, proper sexual prophylaxis, and programs to care for underprivileged communities will immediately contribute to a decline in MRSA incidence and prevalence rates in future generations.
